# Parameter Identification of Fractional-Order Discrete Chaotic Systems

**DOI:** 10.3390/e21010027

**Published:** 2019-01-01

**Authors:** Yuexi Peng, Kehui Sun, Shaobo He, Dong Peng

**Affiliations:** School of Physics and Electronics, Central South University, Changsha 410083, China

**Keywords:** parameter identification, particle swarm optimization, fractional difference, discrete chaotic system

## Abstract

Research on fractional-order discrete chaotic systems has grown in recent years, and chaos synchronization of such systems is a new topic. To address the deficiencies of the extant chaos synchronization methods for fractional-order discrete chaotic systems, we proposed an improved particle swarm optimization algorithm for the parameter identification. Numerical simulations are carried out for the Hénon map, the Cat map, and their fractional-order form, as well as the fractional-order standard iterated map with hidden attractors. The problem of choosing the most appropriate sample size is discussed, and the parameter identification with noise interference is also considered. The experimental results demonstrate that the proposed algorithm has the best performance among the six existing algorithms and that it is effective even with random noise interference. In addition, using two samples offers the most efficient performance for the fractional-order discrete chaotic system, while the integer-order discrete chaotic system only needs one sample.

## 1. Introduction

More than 300 years have passed since Gottfried Leibniz first discussed the concept of fractional calculus. Nevertheless, the proper definition of fractional calculus was not formally established until the 19^th^ Century. Since then, fractional calculus has attracted much attention from researchers. Due to the ability to describe natural phenomena more accurately [1], fractional calculus was introduced to the continuous chaotic system and has been applied in a wide range of fields [2,3,4,5]. However, the research on fractional-order discrete chaotic systems is scarce.

Recently, Edelman [6] proposed a fractional-order standard map and a fractional-order logistic map based on the theory of fractional difference [7]. Owing to this, the fractional-order discrete chaotic system has become a new hot topic [8,9,10,11,12,13,14], especially for its synchronization [11,12,13,14]. Chaos synchronization has great significance in secure communication [15,16,17], and it plays an important role in nonlinear science [18,19,20,21]. Up to now, existing chaos synchronization methods for fractional-order discrete chaotic systems are based on known parameters and fractional orders of an original system [11,12,13,14]. Unfortunately, this is not practical because the parameters in many cases cannot be measured. Thus, parameter identification becomes very important.

Parameter identification of chaotic systems has been studied with different approaches for discrete [22,23,24,25] and continuous [26,27,28,29,30,31,32] systems. In practice, three main methods are utilized, such as the synchronization method, the mathematical solution, and the optimization algorithm. The synchronization method depends on the specific system, so it is difficult to design general updating laws for parameter identification. Meanwhile, because many local optimal solutions are in the landscape of the objective function, the true parameters of the considered system are difficult to identify analytically by the mathematical solution. The optimization algorithm avoids both difficulties [28,29,30,31,32].

The optimization algorithm converts the parameter identification problem into a multidimensional optimization problem that requires some samples and prior knowledge about the structure of the system. Compared with other methods, this approach is easy to implement and not sensitive to the considered system. Thus, optimization algorithms are very popular for parameter identification, including the differential evolution (DE) algorithm [28], a hybrid algorithm that combines particle swarm optimization (PSO) and ant colony optimization (ACO) algorithms [29], the artificial bee colony (ABC) algorithm [30], the bird swarm algorithm (BSA) [31], and the improved hybrid chaotic optimization algorithms [32,33]. However, all of these optimization algorithms were proposed for the study of continuous chaotic systems. As far as the authors know, few people have studied these optimization algorithms for discrete systems, especially for the fractional-order discrete chaotic systems. Thus, it is interesting to study an optimization algorithm for parameter identification of fractional-order discrete chaotic systems. In addition, the choice of the amount of samples used must be considered carefully. Unlike continuous chaotic systems, discrete chaotic systems are very sensitive to the number of samples used for parameter identification. Far fewer samples are needed, relative to the hundreds of samples typically used to identify the parameters of a continuous chaotic system. Because this problem is seldom studied, the problem of sample size is also considered in this paper.

The PSO algorithm is inspired by swarm intelligence [34]. Due to its efficiency and robustness, PSO has already proven that it has better performance than the genetic algorithm (GA), ACO algorithm, and DE algorithm [35] for various optimization problems [36,37,38]. Indeed, many improvements can enhance the optimization effect of PSO, but the results of these efforts are still unsatisfactory in addressing many challenges, like the parameter identification problem. Therefore, an improved PSO algorithm called IPSO is proposed for parameter identification in this paper. To further improve the precision and convergence rate, we proposed a special inertia weight in the IPSO algorithm.

The rest of this paper is organized as follows. In Section 2, the problem of the parameter identification for the discrete chaotic system is described in detail. The IPSO algorithm is presented in Section 3. The numerical simulations are discussed in Section 4, and the paper concludes with a discussion of the results and prospects for further research in Section 5.

## 2. Problem Description

Parameter identification of fractional-order discrete chaotic systems is regarded as an optimization problem [24,25] with the following details. The fractional-order discrete chaotic system is described as:

(1) $$\Delta^{q}X=F\left(X,X_{0},\theta \right),$$

where $X={\left(x_{1},x_{2},\ldots ,x_{M}\right)}^{T}\in R^{M}$ is the *M*-dimensional state vector of the original system and *M* is the sample size. $X_{0}$ is the initial state of original system. $\theta ={\left(\theta_{1},\theta_{2},\ldots ,\theta_{D}\right)}^{T}\in R^{D}$ denotes the original parameter, and *D* is the number of identified parameters. *q* is the fractional order of this discrete chaotic system.

If the structure of the original system is known, then the identified system is described by:

(2) $$\Delta^{\tilde{q}}\tilde{X}=F\left(\tilde{X},X_{0},\tilde{\theta }\right),$$

where $\tilde{X}={\left({\tilde{x}}_{1},{\tilde{x}}_{2},\ldots ,{\tilde{x}}_{M}\right)}^{T}\in R^{M}$ denotes the *M*-dimensional state vector of the identified system. $\tilde{\theta }={\left({\tilde{\theta }}_{1},{\tilde{\theta }}_{2},\ldots ,{\tilde{\theta }}_{D}\right)}^{T}$ is the identified parameter. $\tilde{q}$ is the identified fractional order. On the basis of the measurable *X* and the optimization algorithm, the goal of the parameter identification is defined by:

(3) $$J=\frac{1}{M}\sum_{k=1}^{M}{\left(X_{k}-{\tilde{X}}_{k}\right)}^{2},$$

where *J* is the objective function or the identified error. $X_{k}$ and ${\tilde{X}}_{k}$ (*k* = 1, 2, ..., *M*) denote the state at the $k^{\mathrm{th}}$ time of the original system and the identified system, respectively. This formulation constructs the parameter identification problem as a multidimensional optimization problem. The decision vector is the identified fractional order $\tilde{q}$ and the identified parameter $\tilde{\theta }$. *J* is the objective function that needs to be minimized.

The problem of parameter identification for the fractional-order discrete chaotic system typically presents difficulties for traditional methods. Meanwhile, due to the real-time requirement of system synchronization, the identification method must have the characteristics of high precision and low time consumption. Therefore, our main purpose is to propose a simple, but highly-effective optimization algorithm for parameter identification.

## 3. The Proposed IPSO Algorithm

### 3.1. PSO Algorithm

The PSO algorithm mimics the individual behaviors in a population of birds. Each particle represents a bird, and the algorithm starts with a random initialization of the particle locations. In each iteration, each particle keeps track of its own optimal position and the population’s optimal position to update its position and velocity. The velocity of particle *i* is defined as [34]:

(4) $$V_{i}\left(t+1\right)=\omega V_{i}\left(t\right)+c_{1}r_{1}\left[J_{bi}\left(t\right)-X_{i}\left(t\right)\right]+c_{2}r_{2}\left[J_{g}\left(t\right)-X_{i}\left(t\right)\right],$$

where $V_{i}\left(t\right)$ and $X_{i}\left(t\right)$ are the velocity and position of the particle *i* at the $t^{\mathrm{th}}$ iteration, respectively. ω is called the inertia weight. $c_{1}$ and $c_{2}$ are the learning factors. $r_{1}$ and $r_{2}$ are random numbers between zero and one. $J_{bi}\left(t\right)$ represents the optimal position of the particle *i* after the $t^{\mathrm{th}}$ iteration, and $J_{g}\left(t\right)$ represents the population’s optimal position after the $t^{\mathrm{th}}$ iteration. $X_{i}\left(t\right)$ is the position of particle *i*, and it is defined by:

(5) $$X_{i}\left(t+1\right)=X_{i}\left(t\right)+V_{i}\left(t+1\right).$$

The PSO algorithm is divided into global and local search. The global search enables particles to approach the target quickly, whereas the local search helps all particles finally reach the target.

### 3.2. IPSO Algorithm

In the IPSO algorithm, a special choice of inertia weight is proposed. The inertia weight ω is very important for particle velocities in the PSO algorithm, and it is adjusted according to the particles’ agglomeration [34]. If the particles are dispersed, they need high velocity. Once they gather into a group, the velocities should slow to increase the precision of the algorithm. In general, the particles are dispersed at the beginning of the search, and they form a group as the search goes on. Thus, ω should be set from large to small. Large ω is more favorable for global search, and smaller ω is better for local search. How to set this parameter reasonably is the key problem for the PSO algorithm.

**Figure 1:**
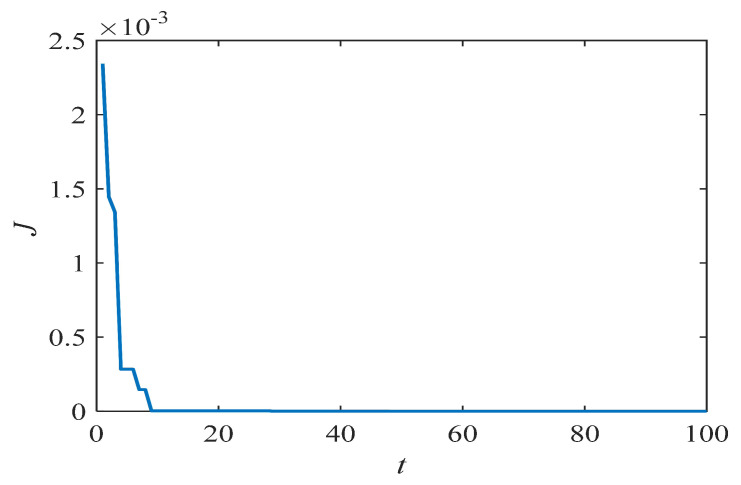
The evolution of *J* in the Hénon map as identified by the PSO algorithm.

**Figure 2:**
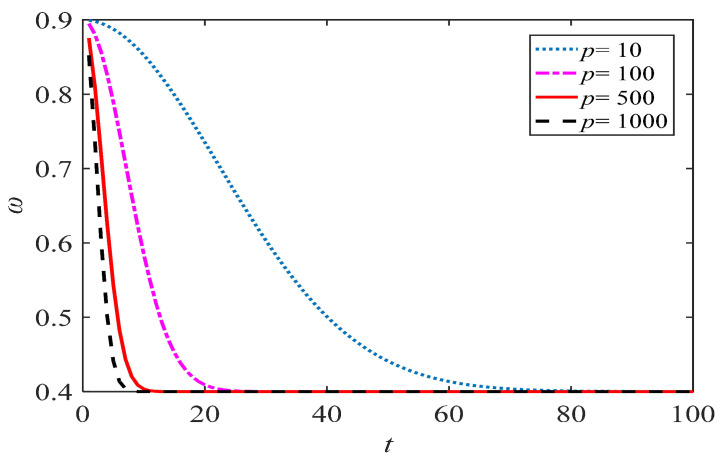
ω with different *p*.

In practice, there are three approaches to modeling the inertia weights. The first one is described as [39]:

(6) $$\omega \left(t\right)=\omega_{max}-\left(\frac{\omega_{max}-\omega_{min}}{T}\right)t,$$

where $\omega_{max}=0.9$ and $\omega_{min}=0.4$. *t* and *T* are the current and maximum iterations of the PSO algorithm, respectively. The second approach is to set ω as a constant (0.7298) [30]. The last approach is the improved inertia weight [37]. However, this kind of inertia weight is not suitable for the problem in this paper, as the results in Section 4.1 demonstrate. When the PSO algorithm is utilized to identify the parameters of the Hénon map, the results are as shown in Figure 1. Similar conclusions are obtained for other discrete chaotic systems. This shows that the algorithm is close to the target after a few iterations, which means that the algorithm spends most of the time on the local search. Therefore, a special ω that is more suitable for the local search is proposed as follows:

(7) $$\omega \left(t\right)=\left(\omega_{max}-\omega_{min}\right)e^{-p{\left(t/T\right)}^{2}}+\omega_{min},$$

where *p* is a constant, and it is set according to what is shown in Section 4.1. ω changes with different *p*-values, as shown in Figure 2. The curve of ω approaches the *y*-axis as *p* increases. However, as *p* grows, the curve changes more slowly. The increase of *p* means that the algorithm will spend more time on the local search, which is helpful for the parameter identification problem in this paper.

### 3.3. Implementation of the IPSO Algorithm

**Figure 3:**
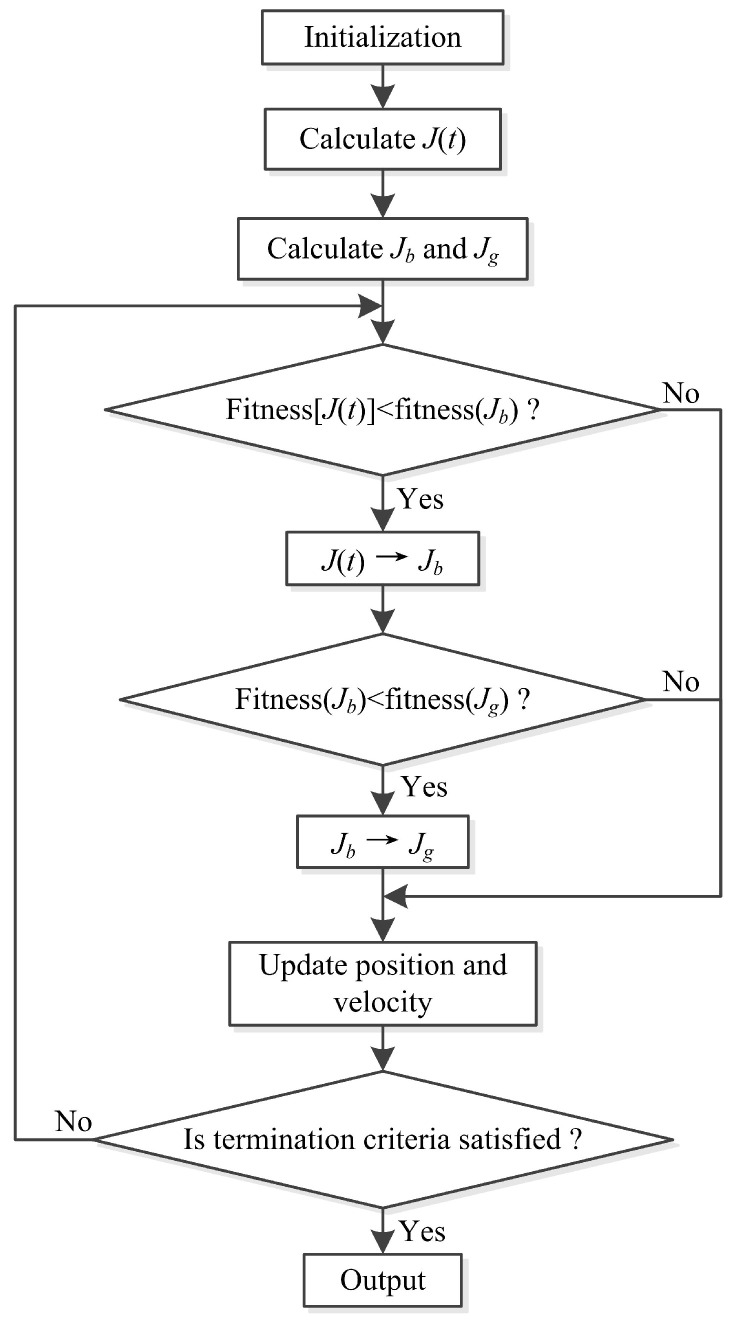
The flowchart of the IPSO algorithm.

The implementation steps of the IPSO algorithm are illustrated in Figure 3 and are summarized as follows:

(a) Initialize each particle in the swarm with random values.

(b) Calculate the fitness value of each particle, and calculate the inertia weight by Equation (7).

(c) Compare the fitness value of each particle with its respective $J_{b}$. If the corresponding fitness value is better than the previous $J_{b}$, then replace $J_{b}$. If the current $J_{b}$ is better than $J_{g}$, then use $J_{b}$ to replace $J_{g}$.

(d) Update the velocity and position of the particle according to Equations (4) and (5).

(e) Repeat Steps (b)–(d) until the termination criterion is satisfied.

## 4. Numerical Simulations

The tests with the numerical simulations discussed in this section are divided into two parts. First, six algorithms are tested for the parameter identification for two typical discrete chaotic systems. The algorithms, include the classical PSO [39], ABC [40], BSA [41], DE [42], APSO [37], and IPSO algorithms. To find the most suitable *p*-value for the IPSO algorithm, *p* was set to 10, 100, 500, and 1000. The Hénon map [43] and the Cat map [44] were the test systems. In the second part, the IPSO algorithm was tested for parameter identification of fractional-order discrete chaotic systems. The problem of selecting the most appropriate number of samples was investigated, and noise was added to test the algorithm’s robustness. The maximum iteration of each algorithm was 100. To eliminate the stochastic nature of the algorithms, we performed over 30 consecutive algorithm runs. Other settings are shown in Table 1. Simulations were based on MATLAB 2016a on an Intel(R) Core(TM) i7-7700HQ CPU @2.80-GHz with 8 GB RAM.

### 4.1. Parameter Identification by Different Optimization Algorithms

The Hénon map is one of the most well-known discrete chaotic systems. It is defined by [43]:

(8) $$\left\{\begin{array}{c} x\left(n+1\right)=1-ax^{2}\left(n\right)+y\left(n\right)\\ y(n+1)=bx(n) \end{array}\right.,$$

where *x* and *y* are state variables. *a* and *b* are system parameters. When (a,b)=(1.4,0.3), the system is chaotic, so we chose these for the following tests. The search ranges were set as 0≤a≤5,0≤b≤5, and they were the same for all algorithms tested. The system was then freely evolved from random initial states, and the first 1000 states was abandoned as the transition process. The sample size was set as M=3.

The Cat map is a discrete chaotic map that has been widely utilized in image encryption [45], and it is defined as [44]:

(9) $$\left\{\begin{array}{c} x(n+1)=\mathrm{mod}(x(n)+ay(n),N)-x(n)\\ y(n+1)=\mathrm{mod}(bx(n)+(ab+1)y(n),N)-y(n) \end{array}\right.,$$

where *x*, *y*, *a*, and *b* have the same meaning as in the Hénon map, mod( , *N*) is the modulo operation, and *N* typically represents the size of the image. N=256 in all trials for this study, and if (a,b)=(1.2,3.4), the system is chaotic. Therefore, we set the original parameters as (a,b)=(1.2,3.4). The search ranges were 0≤a≤5,0≤b≤5.

**Figure 4:**
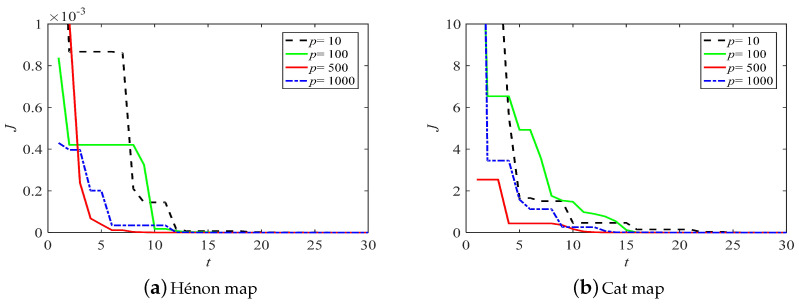
The evolution of *J* obtained by IPSO with different *p*.

The identification results for the Hénon map and the Cat map are shown in Table 2. The APSO algorithm had the highest precision among the ABC, BSA, DE, and PSO algorithms, but it returned larger *J* values than the other four kinds of the IPSO algorithm. Therefore, this demonstrates that the IPSO algorithm had better performance than other five optimization algorithms. Furthermore, the identification precision of IPSO can be increased by increasing *p*. The IPSO algorithm performed optimally with p=500. The evolution of *J* obtained by IPSO with different *p* is shown in Figure 4. When p=500, *J* converged to the objective value after about seven iterations in the Hénon map and twelve iterations in the Cat map, respectively. Therefore, the IPSO (p=500) had the fastest convergence rate of the algorithms. Therefore, p=500 may be a good choice.

In summary, the simulations proved that the IPSO algorithm was more suitable for parameter identification than the other five existing algorithms. This finding lays the foundation for parameter identification of fractional-order discrete chaotic systems.

### 4.2. Number of Samples

The number of samples used had a great effect on the optimization algorithm. Therefore, it was necessary to make a survey of the most appropriate number of samples for the discrete chaotic system.

**Figure 5:**
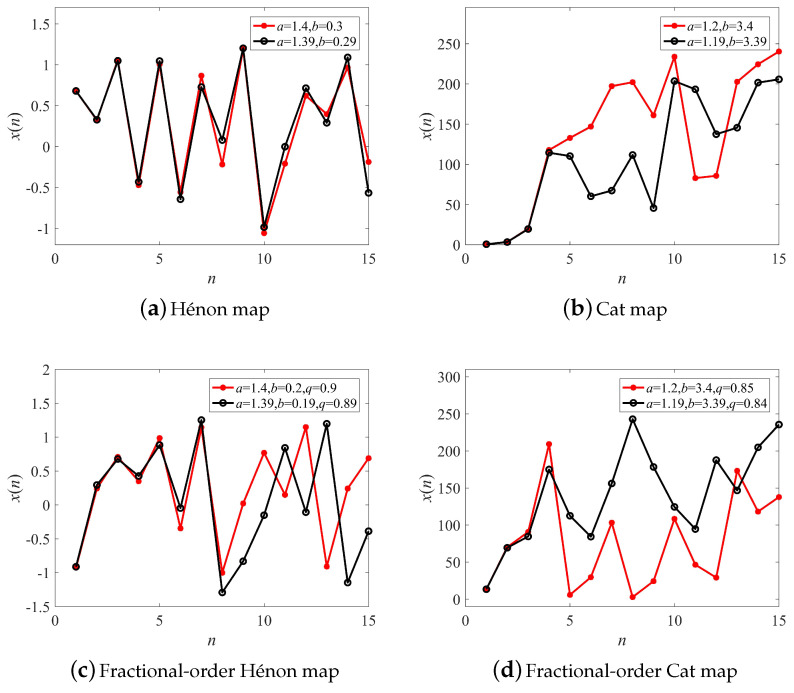
Trajectories of discrete chaotic systems with different parameters.

Since chaotic systems are highly sensitive to variations in initial conditions, their long-term trajectories are impossible to predict from a given set of initial conditions. Trajectories of the Hénon map, the Cat map, and their fractional form with different parameters are illustrated in Figure 5a–d, respectively. When the difference of system parameters was just 0.01, systems produced utterly different trajectories with increasing iterations. Nevertheless, the two trajectories were similar before the iteration n=5, because the behavior of the chaotic system can be predicted in the short-term [46]. Therefore, the following numerical simulations were tested with the number of samples M=1,2,3, and 4, respectively.

Simulation results of the IPSO algorithm for parameter identification with different *M* are presented in Table 3. It shows that the identification precision increased with the decreasing of the sample size. Particularly, one sample was enough to identify the parameters well. Here, less samples not only meant lower time consumption, but also higher precision. This will be a good inspiration for the synchronization and control of discrete chaotic systems based on the optimization algorithm in the future.

### 4.3. Parameter Identification of Fractional-Order Discrete Chaotic Systems

Parameter identification of fractional-order discrete chaotic systems by the IPSO algorithm is investigated in this subsection. According to the above conclusions, simulations were carried out under different sample sizes. The identification precision and time consumption of parameter synchronization were calculated. Finally, parameter identification with random noise is also discussed.

#### 4.3.1. Fractional-Order Hénon Map

**Figure 6:**
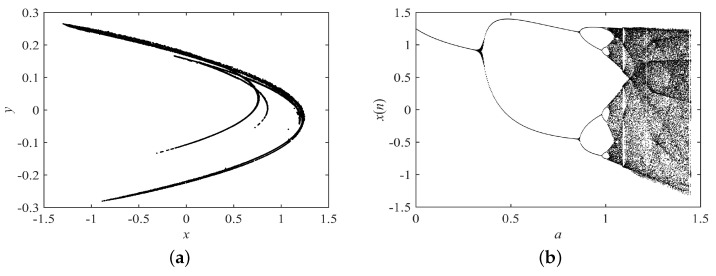
Dynamical behaviors of the fractional-order Hénon map. (**a**) Attractor with a=1.4,b=0.2,q=0.9; (**b**) bifurcation diagram with *a*, and b=0.2,q=0.9.

Fractional-order chaotic maps are usually based on the theory of the fractional difference, which was proposed recently. In this paper, we also use this theory to define the fractional-order Hénon map, and it is described as:

(10) $$\left\{\begin{array}{c} x\left(n+1\right)=x\left(0\right)+\frac{1}{\Gamma (q)}\sum_{j=1}^{n}\frac{\Gamma (n-j+q)}{\Gamma (n-j+1)}\left(1+y\left(j-1\right)-ax^{2}\left(j-1\right)-x\left(j-1\right)\right)\;\\ y\left(n+1\right)=y\left(0\right)+\frac{1}{\Gamma (q)}\sum_{j=1}^{n}\frac{\Gamma (n-j+q)}{\Gamma (n-j+1)}\left(bx\left(j-1\right)-y\left(j-1\right)\right)\; \end{array}\right.,$$

where x(0) and y(0) are the initial values. Γ() is the gamma function, and *q* is the fractional order. *a* and *b* are the system parameters. The dynamical behaviors of the fractional-order Hénon map are shown in Figure 6. The system is chaotic when (a,b,q)=(1.4,0.2,0.9), so we set the original system parameters to (a,b,q)=(1.4,0.2,0.9). Search ranges are set to 0≤a≤5,0≤b≤5,0≤q≤1.

The results of parameter identification by the IPSO algorithm are shown in Table 4. Like in the integer-order system, precision was raised by the decreasing of *M*, and the runtime was also reduced. The precision was not acceptable when M=4; however it improved greatly when *M* was decreased to two or three, and all the parameters can be identified accurately. M=2 is a better choice because it cost less time and had higher precision than M=3. However, the fractional order *q* cannot be identified accurately with M=1, although the obtained *J* was the smallest. In this case, samples were too few to reflect the dynamical behavior of chaotic system. Thus, M=2 was the best choice.

**Figure 7:**
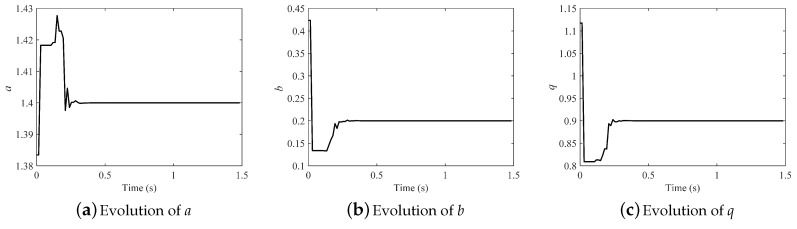
Evolution of parameters in the fractional-order Hénon map.

The evolution of the parameters in the fractional-order Hénon map is illustrated in Figure 7 (M=2). All the parameters can be identified consistently with the original after about only 0.35 s. Results demonstrate that the effect was strong and the convergence rate fast.

The IPSO algorithm was proven to be an effective approach for parameter identification of the fractional-order Hénon map, and it had the highest efficiency with M=2.

#### 4.3.2. Fractional-Order Cat Map

**Figure 8:**
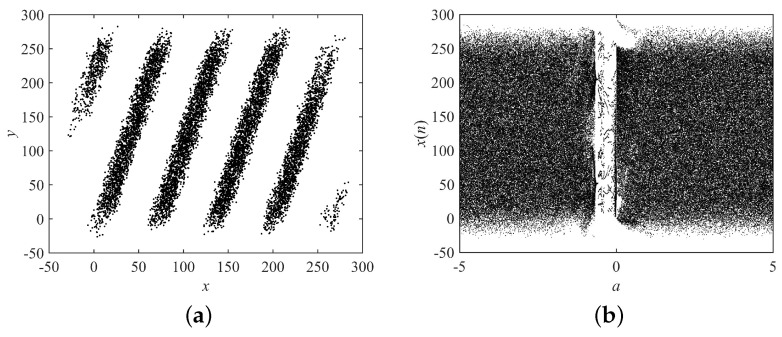
Dynamical behaviors of the fractional-order Cat map. (**a**) Attractor with a=1.2,b=3.4,q=0.85; (**b**) bifurcation diagram with *a*, and b=3.4,q=0.85.

The fractional-order Cat map is defined as:

(11) $$\left\{\begin{array}{c} x\left(n+1\right)=x\left(0\right)+\frac{1}{\Gamma (q)}\sum_{j=1}^{n}\frac{\Gamma (n-j+q)}{\Gamma (n-j+1)}\left(\mathrm{mod}\left(x\left(n\right)+ay\left(n\right),N\right)-x\left(n\right)\right)\\ y\left(n+1\right)=y\left(0\right)+\frac{1}{\Gamma (q)}\sum_{j=1}^{n}\frac{\Gamma (n-j+q)}{\Gamma (n-j+1)}\left(\mathrm{mod}\left(bx\left(n\right)+\left(ab+1\right)y\left(n\right),N\right)-y\left(n\right)\right) \end{array}\right.,$$

where x(0), y(0),a,b,q, and Γ() have the same meaning as in the fractional-order Hénon map, and mod( , *N*) and *N* are the same as in Section 4.1. Th dynamical behaviors of the fractional-order Cat map are shown in Figure 8. When (a,b,q)=(1.2,3.4,0.85), the system was chaotic. Therefore, the original system parameters were set as (a,b,q)=(1.2,3.4,0.85), and the search ranges were set as 0≤a≤5,0≤b≤5,0≤q≤1.

**Figure 9:**
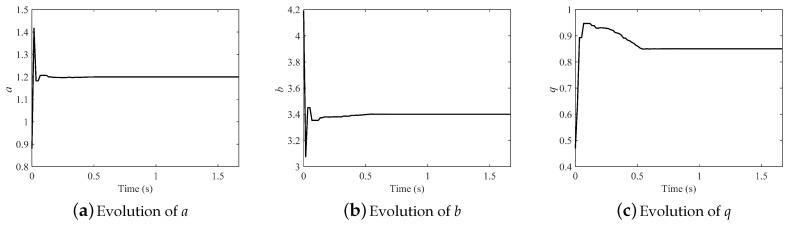
Evolution of parameters in the fractional-order Cat map.

Parameter identification results are listed in Table 5. It seems that the parameters of the fractional-order Cat map were more difficult to identify, because *J* was bigger than that of the fractional-order Hénon map. When M=4, the IPSO algorithm was inapplicable for this system, but the performance improved with decreasing *M*, especially when M=2; all the parameters were identified accurately. As with the fractional-order Hénon map, the *M* still cannot be set to one. The evolution of the parameters in the fractional-order Cat map is illustrated in Figure 9(M=2), and all the parameters were identified accurately after about 0.5 s.

Similar results were found in the fractional-order Cat map. All the above numerical simulations demonstrate the effectiveness of the IPSO algorithm, and M=2 was the most efficient sample size.

#### 4.3.3. Fractional-Order Standard Iterated Map

**Figure 10:**
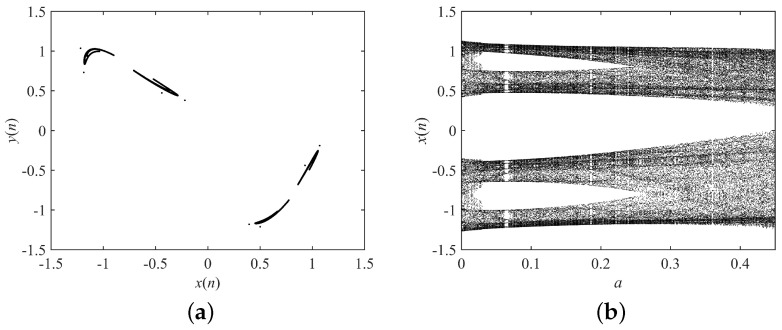
Dynamical behaviors of the fractional-order standard iterated map. (**a**) Attractor with a=0.2,b=0.71,q=0.8; (**b**) bifurcation diagram with *a*, and b=0.71,q=0.8.

Finally, the fractional-order standard iterated map [8], which has some interesting dynamics with hidden strange attractors, is introduced for parameter identification. It is defined as:

(12) $$\left\{\begin{array}{c} x\left(n+1\right)=x\left(0\right)+\frac{1}{\Gamma (q)}\sum_{j=1}^{n}\frac{\Gamma (n-j+q)}{\Gamma (n-j+1)}\left(y\left(j-1\right)-x\left(j-1\right)\right)\\ y\left(n+1\right)=y\left(0\right)+\frac{1}{\Gamma (q)}\sum_{j=1}^{n}\frac{\Gamma (n-j+q)}{\Gamma (n-j+1)}(x\left(j-1\right)+ax^{2}\left(j-1\right)\\ \;\;\;+by^{2}\left(j-1\right)-0.91x\left(j-1\right)y\left(j-1\right)-1.14-y\left(j-1\right)) \end{array}\right.,$$

where x(0), y(0),a,b,q, and Γ() have the same meaning as in the above fractional-order chaotic maps. The dynamical behaviors of the fractional-order standard iterated map with x(0)=0.93,y(0)=−0.44 are shown in Figure 10. When (a,b,q)=(0.2,0.71,0.8), the system was chaotic and had no fixed point. Therefore, the original system parameters were set as (a,b,q)=(0.2,0.71,0.8), and the search ranges were set as 0≤a≤5,0≤b≤5,0≤q≤1.

**Figure 11:**
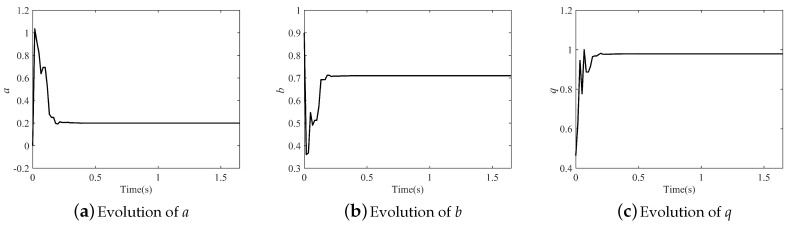
Evolution of parameters in the fractional-order standard iterated map.

Parameter identification results are listed in Table 6. It shows that the parameters of the fractional-order standard iterated map were the most difficult to identify, due to its special properties. When M=2, the IPSO algorithm still obtained the best performance, and it was completely ineffective when M=1. The evolution of parameters in the fractional-order standard iterated map is illustrated in Figure 11(M=2), and all the parameters were identified accurately after about 0.42 s.

In summary, we conclude that the fractional-order standard iterated map was the most difficult to be identified compared to the other two chaotic maps from the results in Table 4, Table 5 and Table 6. In addition, because the fractional-order form had more identified parameters, it was more difficult to identify the parameters compared to those of the integer-order system. M=1 was the most appropriate for parameter identification of the integer-order discrete chaotic system, while the most appropriate sample size of fractional-order discrete chaotic system was M=2.

#### 4.3.4. Parameter Identification under Noise Interference

To test the robustness of the proposed IPSO algorithm, we test parameter identification under random noise in this section. The identified system state vector ${\tilde{x}}_{n}$ (n=1,2,…,M) was changed to:

(13) $${\hat{x}}_{n}={\tilde{x}}_{n}+e\left(2r+1\right),$$

where *e* represents the noise amplitude and *r* denotes a random number (0<r<1). For better comparison, the relative error (RE) is defined as follows:

(14) $$\mathrm{RE}=\frac{|{\tilde{\theta }}_{i}-\theta_{i}|}{\theta_{i}}\times 100\%,$$

where i=1,2,…,D. θ, $\tilde{\theta }$, and *D* are the same as presented in Section 2.

The simulation results are listed in Table 7. All the parameters can be identified correctly without interference from the noise. The maximum REs were 7.80%, 3.27%, and 8.46% at the noise amplitude of 0.1, which means that the parameters in both of the three fractional-order discrete chaotic systems were well reproduced until *e* reached 0.1. Even if e=0.2, the maximum REs of 14.25%, 10.54%, and 14.22% were still acceptable. However, the error grew unacceptably once e=0.3. Consequently, the simulations demonstrated the good performance of the IPSO algorithm for parameter identification with the noise amplitude 0<e<0.2. Moreover, it also shows that the fractional-order standard iterated map was more susceptible to noise than that of the other two fractional-order chaotic maps.

## 5. Conclusions

The problem of parameter identification for fractional-order discrete chaotic systems was addressed with an improved algorithm called IPSO, which includes a special inertia weight. Five existing optimization algorithms were selected for comparison. Simulations were carried out with two typical discrete chaotic systems and three fractional-order discrete chaotic systems. In addition, the selection of the most appropriate sample size and parameter identification under noise interference were also considered. The simulations led to the following three conclusions:

(1) Compared with other algorithms in this paper, namely the ABC, BSA, DE, APSO, and PSO algorithms, IPSO provides the best performance for parameter identification.

(2) For the five systems we tested, sample size M=1 is the most efficient for the integer-order discrete chaotic system, while in the fractional-order discrete chaotic system, M=2 is the best choice. The optimal amount of samples should be quite few because of the chaotic system’s sensitivity to initial conditions.

(3) The IPSO algorithm provides high precision and fast speed for parameter identification of the fractional-order discrete chaotic system, and it is also robust against random noise interference. The limit of acceptable noise is 0<e<0.2.

In the simulation experiments, many factors were set as unknown, such as the fractional order of the system and the parameters, and the noise was included. These simulations were therefore realistic and lay the foundation for practical applications of chaos synchronization, such as image encryption and information security. What is more is that the proposed approach may be useful as a tool for basic research into nonlinear phenomena. We plan to continue this work by applying the proposed algorithm to chaos synchronization and exploring other applications of fractional-order discrete chaotic systems.

## Figures and Tables

**Table 1:** Parameter settings of different optimization algorithms.

Algorithm	Parameter Setting
ABC	NP=40,limit=100
BSA	$N=40,C=S=1.5,a_{1}=a_{2}=1,FQ=3,P\in \left[0.8,1\right],FL\in \left[0.5,0.9\right]$
DE	NP=40,F=0.6,CR=0.6
APSO	$N=40,c_{1}=c_{2}=2,$α = 0.1
PSO	$N=40,c_{1}=c_{2}=2,$ω is calculated according to Equation (6)
IPSO	$N=40,c_{1}=c_{2}=1.4995,$ω is calculated according to Equation (7)

**Table 2:** Comparison of algorithms for parameter identification.

Algorithm	Hénon Map		Cat Map		Rank
	Mean *J*	Best *J*	Mean *J*	Best *J*	
ABC	$5.6690\times 10^{-14}$	$1.2474\times 10^{-15}$	$3.2731\times 10^{-1}$	$2.1897\times 10^{-4}$	9
BSA	$1.0061\times 10^{-16}$	$2.0620\times 10^{-18}$	$6.4257\times 10^{-10}$	$2.4368\times 10^{-14}$	8
DE	$2.1233\times 10^{-16}$	$3.7411\times 10^{-22}$	$7.6605\times 10^{-13}$	$2.2675\times 10^{-15}$	6
APSO	$3.3874\times 10^{-19}$	$5.6299\times 10^{-23}$	$5.2501\times 10^{-16}$	$8.9700\times 10^{-19}$	5
PSO	$4.1570\times 10^{-16}$	$1.2748\times 10^{-20}$	$1.1260\times 10^{-10}$	$4.2450\times 10^{-14}$	7
IPSO (p=10)	$9.6025\times 10^{-24}$	$1.7454\times 10^{-31}$	$3.8624\times 10^{-22}$	$5.2821\times 10^{-26}$	4
IPSO (p=100)	$6.2087\times 10^{-33}$	0	$9.8169\times 10^{-28}$	0	3
IPSO (p=500)	$1.8720\times 10^{-33}$	0	$5.2389\times 10^{-30}$	0	1
IPSO (p=1000)	$4.0058\times 10^{-33}$	0	$8.3194\times 10^{-30}$	0	2

**Table 3:** IPSO algorithm for parameter identification with different *M*.

*M*	Hénon Map		Cat Map	
	Mean *J*	Best *J*	Mean *J*	Best *J*
4	$1.5285\times 10^{-3}$	0	40.3614	0
3	$1.8720\times 10^{-33}$	0	$5.2389\times 10^{-30}$	0
2	$9.4832\times 10^{-34}$	0	$2.2495\times 10^{-31}$	0
1	0	0	$4.1087\times 10^{-34}$	0

**Table 4:** Parameter identification for the fractional-order Hénon map.

*M*	Mean *J* Value	Best *J* Value	Parameter *a*	Parameter *b*	Parameter *q*	Runtime (s)
4	$8.3756\times 10^{-5}$	$3.8138\times 10^{-30}$	1.401329	0.199476	0.896988	1.5690
3	$6.0135\times 10^{-21}$	$7.5496\times 10^{-32}$	1.400000	0.200000	0.900000	1.5322
2	$1.4844\times 10^{-26}$	$9.9378\times 10^{-32}$	1.400000	0.200000	0.900000	1.4840
1	$7.2723\times 10^{-32}$	$9.9234\times 10^{-33}$	1.400000	0.200000	0.849664	1.4169

**Table 5:** Parameter identification for the fractional-order Cat map.

*M*	Mean *J* Value	Best *J* Value	Parameter *a*	Parameter *b*	Parameter *q*	Runtime (s)
4	84.7813	$1.6136\times 10^{-15}$	1.621749	2.918933	0.827996	1.8034
3	$1.7824\times 10^{-05}$	$4.2463\times 10^{-27}$	1.200812	3.400436	0.851201	1.7277
2	$6.3520\times 10^{-12}$	$3.6731\times 10^{-30}$	1.200000	3.400000	0.850000	1.6683
1	$6.4095\times 10^{-30}$	$4.9024\times 10^{-33}$	1.200000	3.400000	0.659461	1.6182

**Table 6:** Parameter identification for the fractional-order standard iterated map.

*M*	Mean *J* Value	Best *J* Value	Parameter *a*	Parameter *b*	Parameter *q*	Runtime (s)
4	9.8521	$1.0785\times 10^{-24}$	0.206732	0.674523	0.975732	1.7177
3	$5.4168\times 10^{-4}$	$2.4652\times 10^{-29}$	0.201029	0.710992	0.980320	1.6636
2	$4.4313\times 10^{-7}$	$6.9847\times 10^{-32}$	0.200062	0.710001	0.978999	1.6499
1	$1.3148\times 10^{-30}$	$3.1456\times 10^{-32}$	2.430401	0.309178	0.741652	1.6203

**Table 7:** Parameter identification under random noise.

Chaotic Map	Parameter	*e* = 0	*e* = 0.05	*e* = 0.1	*e* = 0.2	*e* = 0.3
Fractional-order Hénon map	*a*	1.400000	1.375551	1.412522	1.351937	1.498865
	*b*	0.200000	0.203221	0.215641	0.228513	0.106815
	*q*	0.900000	0.918607	0.908876	0.965400	0.707809
	**Max of RE (%)**	**0.00**	**2.07**	**7.80**	**14.25**	**46.60**
Fractional-order Cat map	*a*	1.200000	1.210705	1.195580	1.164532	1.244084
	*b*	3.400000	3.404629	3.409611	3.422271	3.331411
	*q*	0.850000	0.825809	0.822208	0.939616	0.66823
	**Max of RE (%)**	**0.00**	**2.85**	**3.27**	**10.54**	**25.75**
Fractional-order standard iterated map	*a*	0.199984	0.188551	0.183076	0.228451	0.309276
	*b*	0.710002	0.709989	0.703992	0.670611	0.512843
	*q*	0.979000	0.977320	0.964048	0.996278	0.859164
	**Max of RE (%)**	**0.00**	**5.72**	**8.46**	**14.22**	**54.64**
